# Value-based pricing of brensocatib for frequently exacerbating non-cystic fibrosis bronchiectasis in China: a China-adapted, FEV_1_-inclusive time-dependent markov analysis

**DOI:** 10.3389/fphar.2026.1920335

**Published:** 2026-09-25

**Authors:** Dandan Zhang, Pin Hu, Wei Zhang, Wenwen Zhang

**Affiliations:** Department of Pharmacy, Shanghai Pulmonary Hospital, Tongji University School of Medicine, Shanghai, China

**Keywords:** brensocatib, China, cost-effectiveness, FEV1, markov model, model transferability, non-cystic fibrosis bronchiectasis, value-based pricing

## Abstract

**Objective:**

To estimate a China-specific value-based annual price (P*) for brensocatib 25 mg added to standard care (SC), compared with SC alone, in adults with frequently exacerbating non-cystic fibrosis bronchiectasis, and to assess how strongly the estimate depends on transferred assumptions linking forced expiratory volume in 1 s (FEV_1_) to long-term outcomes.

**Methods:**

A seven-state Markov cohort model with monthly cycles and a lifetime horizon was constructed from the Chinese healthcare system perspective. Chinese sources informed the target population, exacerbation burden, baseline microbiology, background mortality, resource use, costs, discounting, and decision context, while ASPEN supplied the exacerbation rate ratio (0.81) and annual FEV_1_ decline (24 mL with brensocatib 25 mg vs. 62 mL with placebo). In the base case, cohort-mean FEV_1_ was linked to utility and mortality through explicit external mappings. A prespecified structural sensitivity removed both mappings but retained the trial-observed exacerbation benefit. Costs and outcomes were discounted by 4.5% annually. The primary willingness-to-pay (WTP) threshold was CNY 200,000 per quality-adjusted life-year (QALY); uncertainty was examined with a paired 10,000-draw probabilistic sensitivity analysis (PSA).

**Results:**

At an illustrative price of CNY 120,000/year, the FEV_1_-inclusive base case estimated an incremental cost of CNY 551,543, gains of 0.2761 QALYs and 0.3432 discounted life-years, and 0.57 fewer lifetime exacerbations. These results corresponded to an incremental cost-effectiveness ratio of CNY 1,997,665/QALY and P* of CNY 11,692/year. Drug acquisition accounted for almost all of the incremental cost. The PSA median P* was CNY 11,611/year (95% simulation interval CNY 4,145–19,324), and none of 10,000 simulations was cost-effective at CNY 120,000/year for WTP thresholds up to CNY 300,000/QALY. Estimates changed little when China-specific demographic and exacerbation inputs were varied. In contrast, removing the FEV_1_-linked pathway reduced P* to CNY 990/year, while assuming persistent efficacy after attrition increased it to CNY 24,432/year.

**Conclusion:**

With inputs localised wherever credible Chinese evidence was available, the FEV_1_-inclusive base case estimated P* at CNY 11,692/year for a WTP of CNY 200,000/QALY. The broad pricing implication was stable, although the exact estimate depended chiefly on the durability and downstream consequences of the 52-week FEV_1_ difference. Both the base case and the key structural sensitivity remained far below the illustrative annual price of CNY 120,000.

## 1 Introduction

Non-cystic fibrosis bronchiectasis (NCFB) is a chronic airway disorder marked by permanent bronchial dilatation, recurrent infection, neutrophilic inflammation, and pulmonary exacerbations (Aliberti et al., 2021; Polverino et al., 2017; Flume et al., 2018). Patients with frequent exacerbations generally have worse symptoms, greater healthcare use, faster lung-function decline, and a poorer long-term prognosis (Chalmers et al., 2017; Chalmers et al., 2018).

Bronchiectasis imposes a substantial and growing burden in China. A nationwide urban insurance study documented a marked rise in diagnosed prevalence from 2013 to 2017 (Feng et al., 2022). The subsequent BE-China registry described 10,324 patients across 97 centres and found clinical and microbiological profiles that differ from those reported in many Western cohorts (Xu et al., 2025). Differences in patient characteristics, pathogen distribution, resource use, unit costs, mortality, and decision thresholds make foreign economic evaluations difficult to transfer directly to the Chinese setting.

Brensocatib is an oral, reversible dipeptidyl peptidase-1 inhibitor that reduces activation of neutrophil serine proteases. The phase II WILLOW and phase III ASPEN trials showed reductions in pulmonary exacerbations (Chalmers et al., 2020; Chalmers et al., 2025), while a Chinese phase II trial of another DPP-1 inhibitor provided contextual support for the mechanism in China (Zhong et al., 2025). In ASPEN, the annualised decline in forced expiratory volume in 1 s (FEV_1_) at week 52 was 24 mL with brensocatib 25 mg and 62 mL with placebo, a between-group difference of 38 mL/year (95% CI 11–65) (Chalmers et al., 2025). Brensocatib received US Food and Drug Administration approval in August 2025 (Babar et al., 2025). We estimated a China-adapted value-based annual price using an FEV_1_-inclusive base case, accompanied by a prespecified structural sensitivity in which the FEV_1_-linked pathway was disabled. Inputs were localised where credible Chinese evidence was available; the remaining transferred assumptions were made explicit and tested.

## 2 Methods

### 2.1 Analytical framework and evidence

The study was conducted in accordance with the Consolidated Health Economic Evaluation Reporting Standards 2022 (CHEERS 2022) (Husereau et al., 2022) and the China Guidelines for Pharmacoeconomic Evaluations 2025 (Wu and Liu, 2025). The model took the Chinese healthcare-system perspective, included direct medical costs in 2025 CNY, used monthly cycles over a lifetime horizon, and discounted costs and health outcomes at 4.5% per year. The primary willingness-to-pay (WTP) threshold was CNY 200,000 per quality-adjusted life-year (QALY), approximately twice China’s 2025 *per capita* gross domestic product (National Bureau of Statistics of China, 2026); scenarios covered CNY 50,000–300,000/QALY.

The cohort Markov framework projects outcomes beyond trial follow-up by moving the modelled population among predefined health states over repeated cycles. Health effects were expressed as quality-adjusted life-years (QALYs), which combine survival and health-related quality of life; one QALY represents 1 year in full health. Cost-effectiveness was summarised by the incremental cost-effectiveness ratio (ICER), calculated as the additional cost per additional QALY for brensocatib versus standard care and assessed against the willingness-to-pay threshold. Supplementary Sections S1.2–S1.3, S2, S8 describe the parameter-oriented search, evidence basis, and full input specifications.

The target population comprised adults with NCFB who had experienced at least two exacerbations in the previous 12 months (Chalmers et al., 2025). Prespecified model-entry values were age 62 years and 56.7% female, close to but not identical to the overall BE-China cohort (mean age 63.1 years; 58.2% female) (Xu et al., 2025). Chinese sources were prioritised for population characteristics, disease burden, baseline microbiology, mortality, resource use, costs, discounting, and the decision context; randomised trials supplied relative treatment effects. Supplementary Sections S1, S2, S8 describe the parameter-oriented search, evidence hierarchy, and full input specifications.

The PubMed/MEDLINE and Scopus searches returned 1,056 records, leaving 752 after deduplication. Two reviewers independently screened titles and abstracts and then full texts, resolving disagreements by discussion. Of 313 reports assessed in full, 8 were retained for parameter extraction. Database searching was supplemented by hand-searches of pivotal trials, reference lists, regulatory and health technology assessment documents, guidelines, and national statistics. Supplementary Sections S1.2 provides the search strategies, eligibility criteria, selection flow, and reasons for full-text exclusion.

### 2.2 Model structure

The time-dependent cohort Markov model contained seven clinical states: stable NCFB and exacerbation states without chronic infection; corresponding stable and exacerbation states with chronic *Pseudomonas aeruginosa* (PA) or nontuberculous mycobacterial infection (NTM); and death. Each exacerbation onset contributed one event count, one cost, and one QALY decrement. Treatment retention, washout, and forced expiratory volume in 1 s (FEV_1_) trajectories were tracked in internal layers. BE-China reported PA and NTM positivity using different tested denominators, so the reference model pragmatically treated 20.8% and 5.6% as mutually exclusive baseline weights rather than directly observed whole-cohort prevalences (Xu et al., 2025). PA/NTM acquisition and clearance were introduced only in exploratory scenarios (Araújo et al., 2018; Conceição et al., 2024; Kwak et al., 2019). Acute QALY decrements were applied as fixed absolute losses by severity and were not rescaled to the lower background utility assigned to PA- or NTM-positive states (Starkie et al., 2011; Camac et al., 2021). The model structure is shown in Figure 1.

**FIGURE 1:**
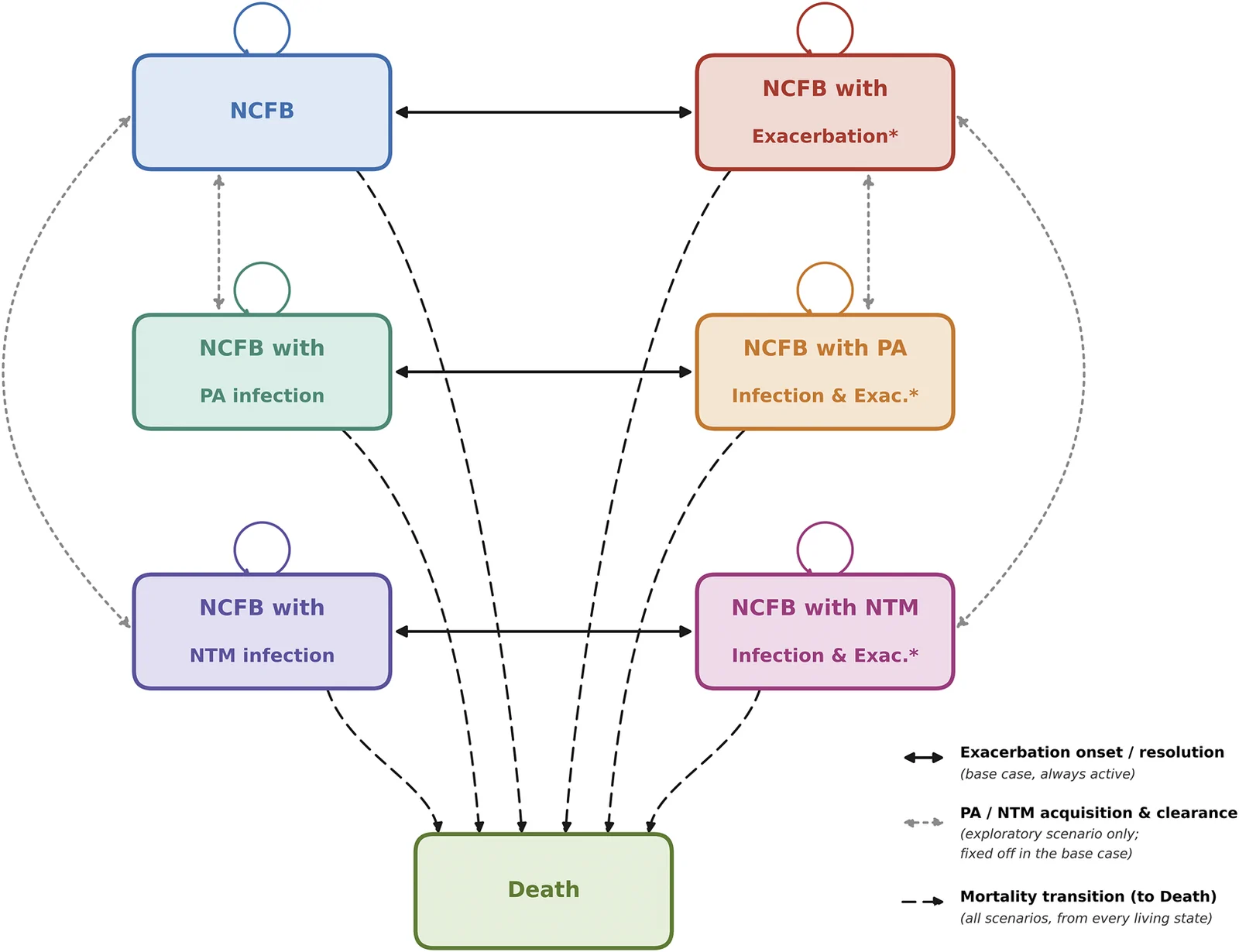
Seven-state clinical model and the FEV_1_-inclusive base-case pathway. Solid black arrows show exacerbation onset and resolution within each infection stratum (stable NCFB ↔ NCFB with exacerbation; PA infection ↔ PA infection with exacerbation; NTM infection ↔ NTM infection with exacerbation); these transitions operate in every analysis. Fine grey dashed arrows represent PA and NTM acquisition and clearance. Because the reference structure fixes PA/NTM status using pragmatic baseline weights from culture-positive subsets, those transitions are inactive in the base case and enabled only in the prespecified exploratory scenario. PA and NTM are treated as mutually exclusive chronic infections; the model includes neither direct PA↔NTM switching nor a combined acquisition-and-exacerbation transition. Black dashed arrows show mortality from each living state. The base case combines the trial-observed reduction in exacerbations with ASPEN 25-mg lung-function preservation and explicit FEV_1_-to-utility and FEV_1_-to-mortality mappings. The key structural sensitivity removes the FEV_1_-linked pathway but retains the same exacerbation effect. Each exacerbation is counted and costed once, at onset. Source: authors’ model design, informed by the cited clinical and epidemiological evidence.

Age- and sex-specific all-cause mortality rates from the 2025 China Health Statistics Yearbook (National Health Commission of the People’s Republic of China, 2025) were reconstructed and updated monthly as the cohort aged. The resulting age pattern was cross-checked against national mortality trends reported by Ling et al. (Ling et al., 2024). An NCFB standardised mortality ratio (SMR) of 2.0, informed jointly by BE-China and Loebinger et al. (Xu et al., 2025; Loebinger et al., 2009), was applied uniformly to all living states. To limit double counting, the reference structure did not add separate mortality multipliers for acute exacerbations or infection states. Supplementary Sections S2, S3, S6, S7 provide the full transition logic, mortality reconstruction, exacerbation calibration, and infection assumptions.

### 2.3 Treatment effects, costs, and structural assumptions

ASPEN provided the 25-mg exacerbation rate ratio (0.81; 95% confidence interval [CI] 0.69–0.94) and week-52 FEV_1_ changes (−24 mL with brensocatib and −62 mL with placebo; between-group difference 38 mL, 95% CI 11–65) (Chalmers et al., 2025). In the FEV_1_-inclusive base case, cohort-mean FEV_1_ was mapped to utility using the relationship reported by Rutten-van Molken et al. (Rutten-van Mölken et al., 2006) and to mortality using the bronchiectasis relationship reported by Loebinger et al. (Loebinger et al., 2009). The broader natural-history scaffold was adapted from the COPD simulation model developed by Borg et al. (Borg et al., 2004). The prespecified key structural sensitivity removed both FEV_1_ mappings but otherwise retained the trial-observed exacerbation effect, treatment exposure, costs, and model structure. The FEV_1_ mortality multiplier was normalised to 1.0 at model entry (FEV_1_ 1.81 L; 71.0% predicted) and increased only with cumulative decline from that baseline. It was then multiplied by the common NCFB SMR rather than anchored to 100% predicted.

Trial non-completion at 52 weeks (13.3%) served as a pragmatic annual attrition proxy (Chalmers et al., 2025). In the retained-only reference case, drug costs and new treatment benefit stopped at attrition; subsequent FEV_1_ decline reverted to the standard-care (SC) rate, while the separation already accumulated was retained. Alternative scenarios allowed persistent post-attrition efficacy, six- or 12-month washout, or waning exacerbation efficacy. Chinese studies informed stable-disease and exacerbation costs (Ma et al., 2020; Lei et al., 2024); chronic PA/NTM cost increments came from external sources (Blanchette et al., 2017; Marras et al., 2018) and were treated as uncertain. The reference case assigned no net adverse-event cost or QALY loss, with a conservative burden tested separately. CNY 120,000/year was an illustrative analytical price, not a mainland-China acquisition price observed in the inputs. All parameter values and distributions are reported in the Supplementary Table S4. In the 6- and 12-month washout scenarios, the exacerbation RR and future FEV_1_ slope benefit declined linearly, but the FEV_1_ separation already accumulated was retained. In the persistent-after-attrition scenario, billing stopped while both effects continued indefinitely among surviving former users.

### 2.4 Analyses, uncertainty, and validation

The ICER was calculated as incremental cost divided by incremental QALYs. The value-based annual price was obtained from P* = (WTP × ΔQ − ΔCother)/D, where ΔQ denotes incremental QALYs, ΔCother incremental non-drug cost, and D discounted retained-treatment person-years. Uncertainty was examined through deterministic and structural analyses and a paired 10,000-draw probabilistic sensitivity analysis. Base-case expected value of perfect information (EVPI) and grouped expected value of partial perfect information (EVPPI) were also estimated (Strong et al., 2014). Validation covered transition-matrix conservation, event accounting, treatment-retention bookkeeping, mortality and FEV_1_ trajectories, agreement between independent implementations, and a nested test in which disabling both FEV_1_ mappings exactly reproduced the FEV_1_-disabled model (Eddy et al., 2012). Supplementary Sections S9–S12 provide further analytical and validation details.

## 3 Results

### 3.1 Base-case outcomes and treatment persistence

At the illustrative annual price of CNY 120,000, the FEV_1_-inclusive base case produced an incremental cost of CNY 551,543, a gain of 0.2761 QALYs, and 0.3432 additional discounted life-years. Lifetime exacerbations fell from 19.46 to 18.89, a reduction of 0.57, while the ICER was CNY 1,997,665/QALY. At a WTP of CNY 200,000/QALY, the corresponding value-based annual price was CNY 11,692. Drug acquisition contributed CNY 549,905 of the incremental cost. Exacerbation costs decreased by CNY 1,643, but longer survival raised stable-disease and chronic-infection management costs, leaving a small net increase of CNY 1,639 in non-drug costs (Table 1).

**TABLE 1:** Disaggregated FEV_1_-inclusive base-case results at CNY 120,000/year (2025 CNY)**.** Costs, QALYs, and life-years are discounted; lifetime exacerbations are undiscounted. Totals may differ slightly from the sum of components because of rounding.

Component/outcome	Brensocatib + SC	SC	Incremental
Drug acquisition	549,905	0	549,905
Stable disease management	42,653	41,164	1,489
Chronic PA management	36,803	35,518	1,285
Chronic NTM management	14,532	14,025	507
Exacerbation events	27,839	29,481	−1,643
Total discounted cost	671,731	120,188	551,543
QALYs	6.2999	6.0238	0.2761
Discounted life-years	9.8298	9.4866	0.3432
Lifetime exacerbations	18.89	19.46	−0.57

Source: authors’ model calculations.

The smaller lifetime reduction in exacerbations arises because longer modelled survival leaves patients at risk for longer; it does not reflect weakening of the trial-based rate ratio. With the FEV_1_-linked survival pathway disabled, the model generated no survival gain but avoided 1.64 lifetime exacerbations; incremental QALYs fell to 0.0093 and P* to CNY 990/year. Six- and 12-month washout raised P* only slightly, to CNY 12,106 and CNY 12,507, whereas persistent efficacy after attrition raised it to CNY 24,432 (Table 2).

**TABLE 2:** Base-case, key structural sensitivity, and treatment-persistence results.

Scenario	ΔQALY	ΔLY	Exacerbations avoided	P*(CNY/year)	ICER (CNY/QALY) at CNY 120,000/year
FEV_1_-inclusive base case	0.2761	0.3432	0.57	11,692	1,997,665
FEV_1_-linked outcomes disabled	0.0093	0	1.64	990	60,142,140
Six-month linear washout	0.286	0.3554	0.6	12,106	1,929,592
Twelve-month linear washout	0.2956	0.3673	0.61	12,507	1,867,852
Persistent efficacy after attrition	0.5807	0.7312	1.82	24,432	960,496

Source: authors’ model calculations.

### 3.2 Deterministic sensitivity analysis

Among the one-way analyses, the ASPEN FEV_1_-preservation difference had the greatest influence on the base-case value-based price. Across its 95% CI (11–65 mL/year), P* ranged from CNY 4,046 to CNY 18,615. Discounting (CNY 11,226–17,385), the FEV_1_ mortality mapping (CNY 10,336–12,638), stable utility, and the FEV_1_ utility mapping were the next most influential inputs. The exacerbation rate ratio remained clinically important, but it changed P* less than the lung-function and outcome-mapping parameters. Once FEV_1_-linked outcomes were removed, the exacerbation rate ratio became the main driver: P* ranged from CNY 313/year at RR 0.94 to CNY 1,615/year at RR 0.69 (Supplementary Table S9). The one-way sensitivity results are shown in Figure 2.

**FIGURE 2:**
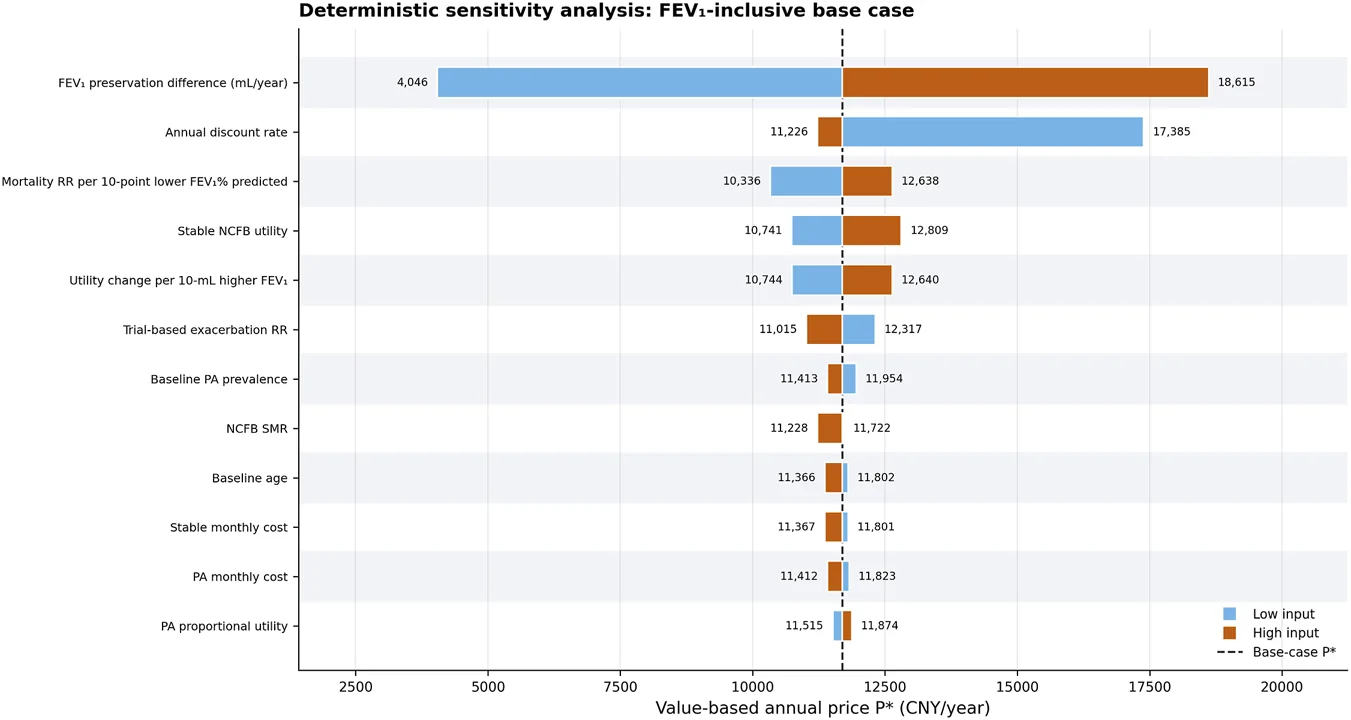
One-way deterministic sensitivity analysis of P* in the FEV_1_-inclusive base case at WTP CNY 200,000/QALY. The ASPEN FEV_1_-preservation difference has the largest effect, followed by discounting and the FEV_1_ mortality and utility mappings. Source: authors’ model calculations.

### 3.3 Probabilistic sensitivity analysis

In the 10,000 paired simulations, mean incremental QALYs and life-years were 0.2790 and 0.3441 in the base case. At CNY 120,000/year, the ratio-of-means ICER was CNY 1,998,232/QALY. Median P* was CNY 11,611/year (95% simulation interval CNY 4,145–19,324). No draw was cost-effective at the illustrative price under WTP thresholds of CNY 100,000, CNY 200,000, or CNY 300,000/QALY, giving an empirical probability of 0% at the simulation resolution of 0.01%. In the paired FEV_1_-disabled sensitivity, median P* was CNY 954/year (95% simulation interval CNY 267–1,849); the base-case P* was higher in 99.73% of paired draws. The joint PSA results are shown in Figure 3.

**FIGURE 3:**
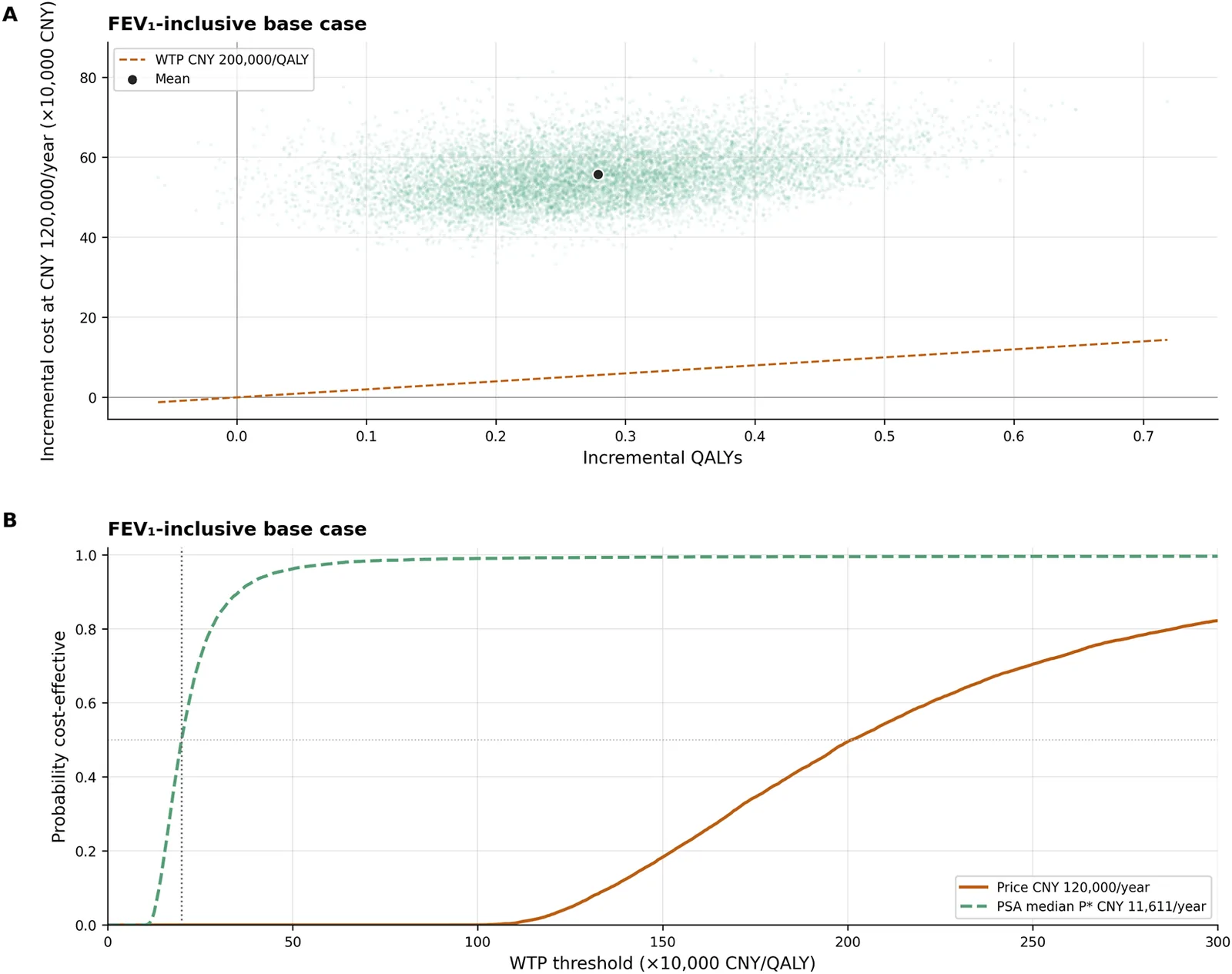
Probabilistic sensitivity analysis for the FEV_1_-inclusive base case (10,000 draws; seed 42). Panel **(A)** presents the cost-effectiveness plane at CNY 120,000/year. Panel **(B)** compares the cost-effectiveness acceptability curves (CEACs) at the illustrative price and the PSA-median value-based price. Source: authors’ probabilistic model calculations.

### 3.4 Localised clinical inputs and structural assumptions

Changes to China-specific baseline inputs had little effect on P*. Varying the annual exacerbation rate from 1.29 to 2.01, starting age from 60 to 65 years, or the female proportion to the 49.0% general-population share reported in the 2025 national statistical communiqué (National Bureau of Statistics of China, 2026) kept P* within CNY 11,366–11,897 (Figure 4; Supplementary Table S8). Baseline infection-state scenarios ranged more widely, from CNY 7,780 for the NTM-positive subgroup to CNY 12,918 for the infection-negative subgroup. The spread reflects differences in background utility, chronic-management costs, and exacerbation burden rather than evidence of validated treatment-effect modification. Because the reference structure applies the same SMR to all living states, infection status did not change survival (ΔLY = 0.3432 in every subgroup; Figure 5). Assumptions without direct Chinese long-term evidence mattered more: removing FEV_1_-linked outcomes reduced P* to CNY 990, persistent efficacy after attrition raised it to CNY 24,432, and the optimistic dynamic-infection scenario yielded CNY 13,516. All deterministic estimates remained below the illustrative annual price of CNY 120,000.

**FIGURE 4:**
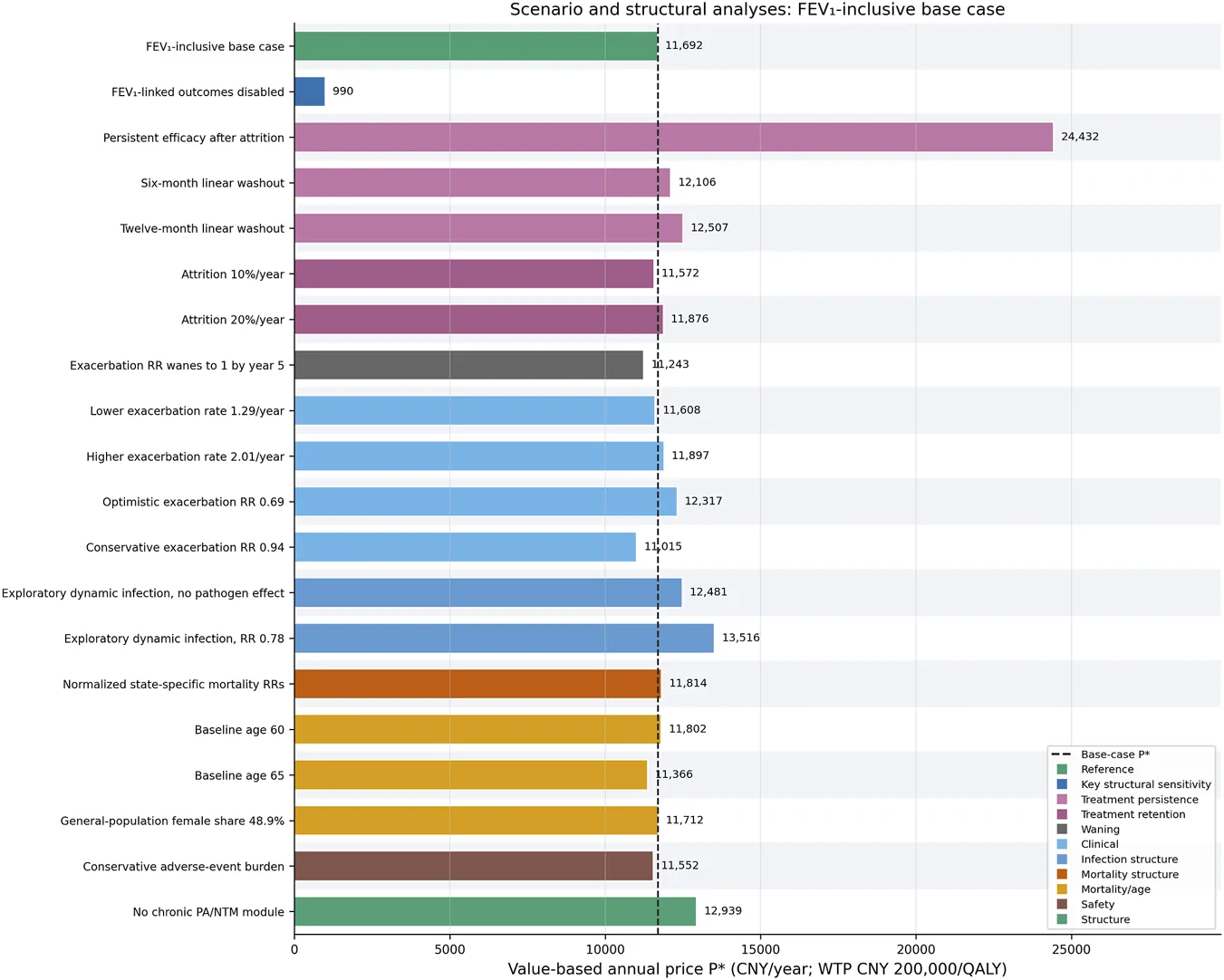
Scenario and structural analyses for the FEV_1_-inclusive base case. The analysis without FEV_1_-linked outcomes is the prespecified key structural sensitivity. All displayed values remain below the illustrative annual price of CNY 120,000. Source: authors’ model calculations.

**FIGURE 5:**
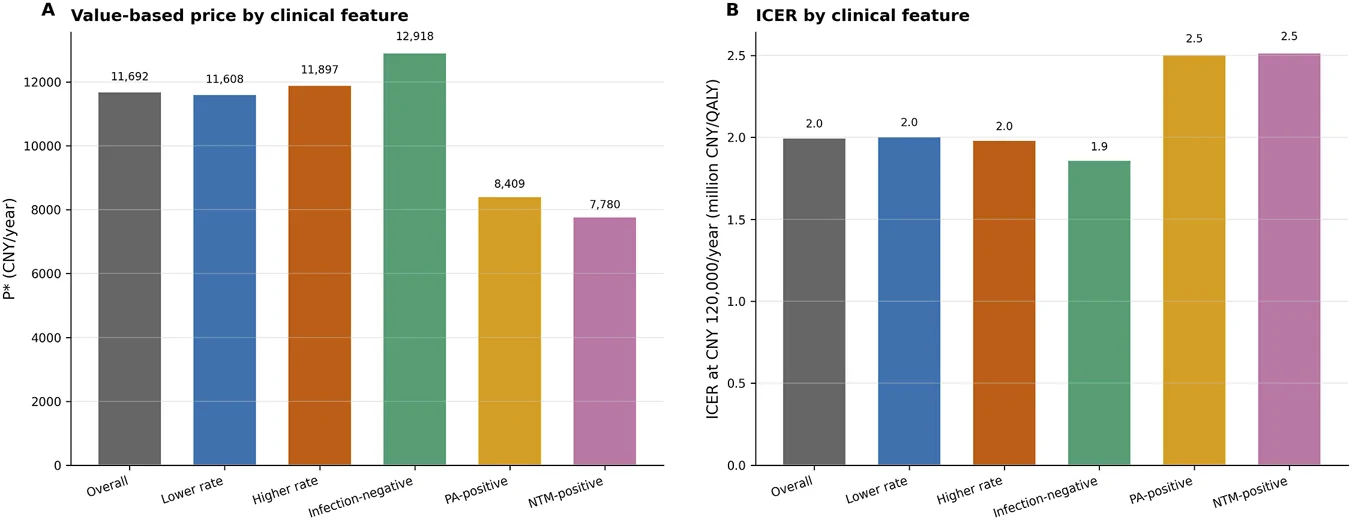
Deterministic clinical-feature scenarios for the FEV_1_-inclusive base case. The scenarios vary event-rate calibration or baseline infection state while holding the relative treatment effect constant; they do not identify validated treatment-effect subgroups. Source: authors’ model calculations. **(A)** Value-based price by clinical feature. **(B)** ICER by clinical feature.

### 3.5 Threshold pricing

At the primary WTP threshold of CNY 200,000/QALY, base-case P* was CNY 11,692/year, or 9.74% of the illustrative CNY 120,000 price. P* rose approximately linearly with WTP, from CNY 2,655/year at CNY 50,000/QALY to CNY 17,717/year at CNY 300,000/QALY (Figure 6; Supplementary Sections S9). At CNY 120,000/year, the base-case ICER was about 10.0 times the primary WTP threshold, and the illustrative price was 10.3 times the deterministic P*. The FEV_1_-disabled P* at the primary threshold was CNY 990/year.

**FIGURE 6:**
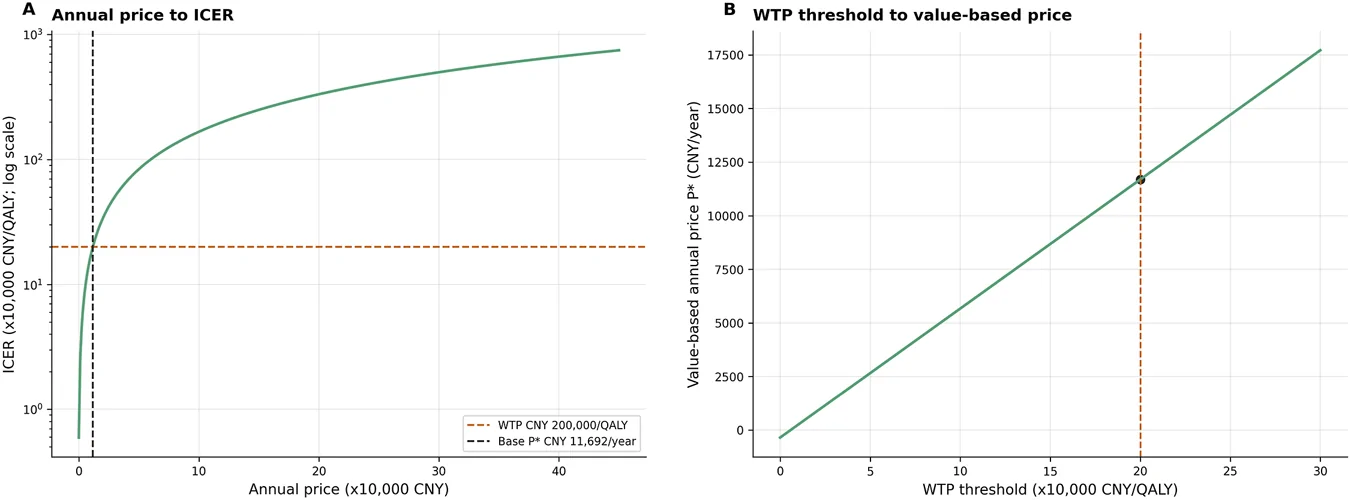
Threshold-pricing analysis for the FEV_1_-inclusive base case. Panel A relates annual price to the ICER; Panel B relates WTP to P*. Source: authors’ model calculations. **(A)** Annual price to ICER. **(B)** WTP threshold to value-based price.

### 3.6 Validation and value of information

Transition-matrix row sums were exact within machine precision, and each event cost and QALY loss was charged once at onset. At month 12, 86.7% of surviving patients remained on treatment. Median survival in the base case was 13.38 years with SC and 14.16 years with brensocatib. Cohort-mean FEV_1_ was 1.748 versus 1.784 L at month 12 and 1.500 versus 1.637 L at month 60. The independent implementations agreed to numerical precision, with a maximum absolute difference below 3 × 10^−9^. Disabling both FEV_1_ mappings within the base-case program reproduced the independent FEV_1_-disabled result exactly (maximum absolute error 0), confirming that the exacerbation-only pathway contained no residual FEV_1_ effect.

At CNY 120,000/year, every PSA draw favoured SC, giving an empirical per-person EVPI of CNY 0 in the finite simulation sample. This result does not indicate an absence of parameter uncertainty. EVPI instead peaked near the probabilistic decision price of CNY 11,691/year, reaching CNY 7,432/person. The partial EVPPI pattern in Figure 7 (Panel B) was dominated by the FEV_1_ treatment effect (CNY 5,313). Smaller values were estimated for event disutility and other utilities (CNY 968), the FEV_1_ outcome mapping (CNY 953), exacerbation efficacy and rates (CNY 533), direct medical costs (CNY 295), baseline infection composition (CNY 235), treatment attrition (CNY 139), and mortality (CNY 98). Because baseline infection composition and the FEV_1_ parameters were sampled in the base-case PSA, each produced a non-zero partial EVPPI; these groups are not additive. In the FEV_1_-disabled sensitivity, EVPI at its decision price was approximately CNY 744/person.

**FIGURE 7:**
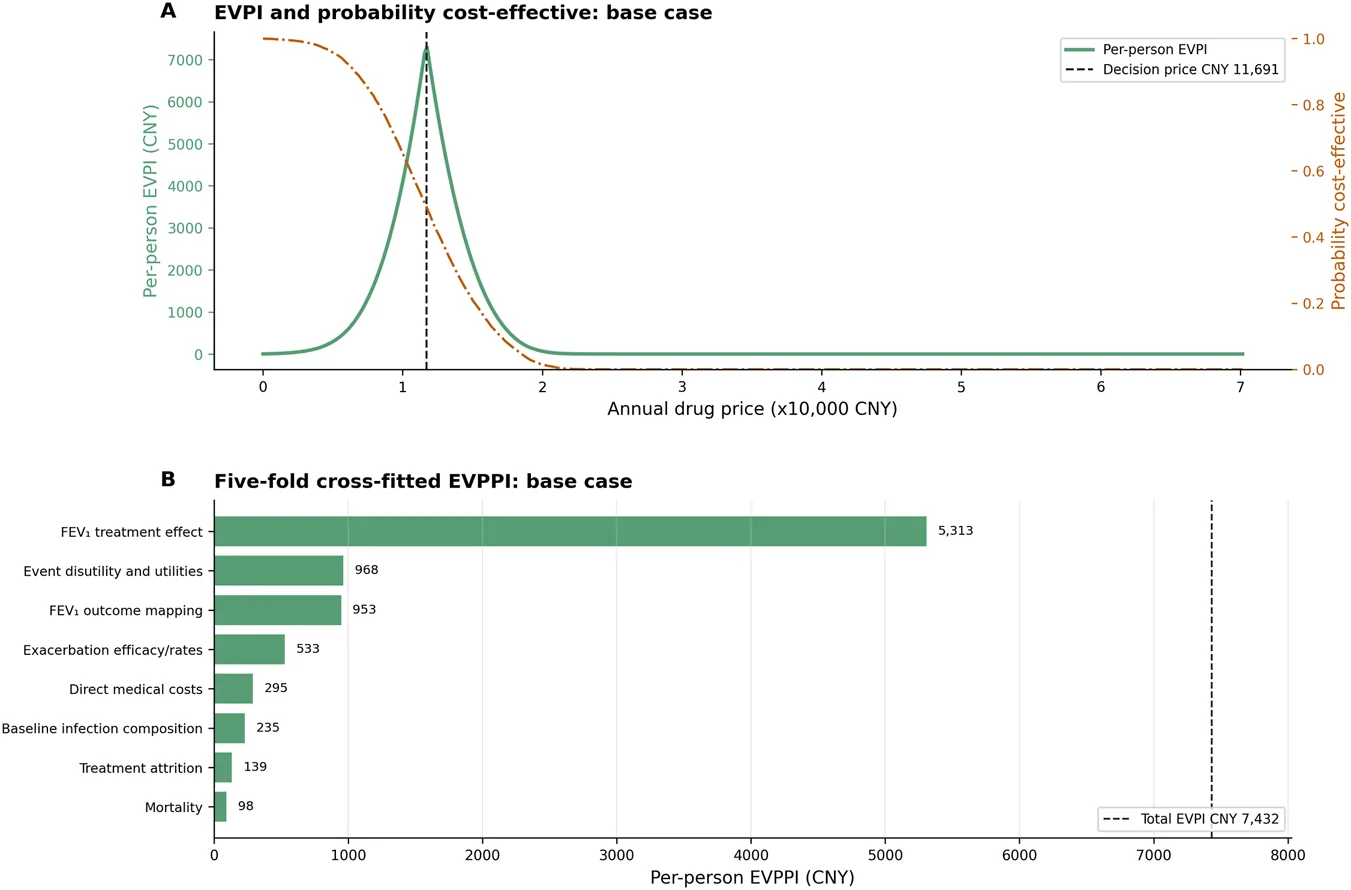
Value-of-information analysis for the FEV_1_-inclusive base case at WTP CNY 200,000/QALY. Panel A shows EVPI and the probability of cost-effectiveness around the decision price; Panel B reports regression-based EVPPI estimates by parameter group. Source: authors’ model calculations. **(A)** EVPI and probability cost-effective: base case. **(B)** Five-fold cross-fitted EVPPI: base case.

## 4 Discussion

### 4.1 Principal findings

At the illustrative annual price of CNY 120,000, brensocatib was not cost-effective in the FEV_1_-inclusive base case. Gains of 0.2761 QALYs and 0.3432 discounted life-years corresponded to an ICER of approximately CNY 2.0 million/QALY and a value-based price of CNY 11,692/year. No prespecified scenario approached CNY 120,000/year. The size of the estimate was nevertheless structurally sensitive: removing the FEV_1_-to-utility and FEV_1_-to-mortality mappings lowered P* to CNY 990/year, whereas persistent efficacy after attrition raised it to CNY 24,432/year. The evidence therefore supports a much lower price than the illustrative benchmark, while leaving uncertainty about the exact value within that range.

The reduction of 0.57 lifetime exacerbations in the FEV_1_-inclusive base case should not be read as attenuation of the trial-based rate ratio. Longer modelled survival kept treated patients at risk for exacerbations for more time; once the FEV_1_-linked survival benefit was removed, 1.64 exacerbations were avoided. Almost all incremental cost came from drug acquisition. Non-drug costs rose slightly because the survival gain extended routine and chronic-infection care. This combination explains why a clinically meaningful relative reduction in exacerbations did not support a high value-based price at the illustrative drug cost.

### 4.2 Localisation, transferability, and comparison with previous evaluations

The model distinguished between inputs that could be localised credibly and those that still had to be transferred. Chinese registries, national statistics, cost studies, and pharmacoeconomic guidance informed the target population, baseline age and sex, exacerbation burden, PA/NTM baseline weights, background mortality, stable-disease and hospital costs, discounting, and the decision context (Feng et al., 2022; Xu et al., 2025; Wu and Liu, 2025; National Bureau of Statistics of China, 2026; National Health Commission of the People’s Republic of China, 2025; Ling et al., 2024; Ma et al., 2020; Lei et al., 2024). Relative treatment effects came from ASPEN; SAVE-BE was used only as contextual evidence because it evaluated a different DPP-1 inhibitor (Chalmers et al., 2025; Zhong et al., 2025). On that basis, the model is more accurately described as China-adapted than fully localised.

For inputs that lacked adequate Chinese evidence, particularly the FEV_1_ outcome mappings, long-term efficacy after attrition, chronic PA/NTM management costs, and infection natural history, the best available external evidence was retained and its provenance identified. Structural scenarios, deterministic sensitivity analysis, paired PSA, and value-of-information analysis were then used to show how much these choices mattered. The distinction had practical consequences: varying local demographic and exacerbation inputs left P* close to the base case, whereas removing FEV_1_-linked outcomes or allowing efficacy to persist after attrition produced estimates spanning CNY 990 to CNY 24,432/year. This range does not show that the transferred inputs are invalid; it shows how strongly the pricing estimate depends on accepting them.

The result is broadly consistent with US economic evaluations, which likewise found that brensocatib would need a substantial price reduction to meet conventional cost-effectiveness thresholds (Wasfy et al., 2025; McKenna et al., 2026). The ICER assessment placed its health-benefit price benchmark at US$3,100–3,700/year and treated the lung-function effect as a scenario rather than the primary case (Wasfy et al., 2025). A direct currency conversion would not be informative because the decision thresholds, costs, epidemiology, and model structures differ. The present analysis shows instead that the broad price-value conclusion persists after adaptation to China, while isolating the additional value generated through the FEV_1_ pathway.

### 4.3 Policy and research implications

At WTP CNY 200,000/QALY, the deterministic value-based price was 9.7% of the illustrative annual price of CNY 120,000. These figures are model-based decision benchmarks, not reimbursement quotations. A managed-entry or outcomes-based agreement could help resolve uncertainty about treatment durability, discontinuation, and FEV_1_ trajectories. It would be most informative after the negotiated price moved closer to the modelled range, because additional evidence cannot by itself bridge an approximately ten-fold price gap.

At CNY 120,000/year, empirical EVPI was zero because every probabilistic draw favoured SC; near the decision price, it rose to CNY 7,432/person. Further research becomes more relevant once the price approaches the modelled value range. The most consequential evidence gaps are discussed below.

### 4.4 Limitations

The treatment-effect pathway is the main structural uncertainty. The base case extrapolated a 52-week between-arm FEV_1_ slope difference, used cohort-mean rather than patient-level trajectories, and retained accumulated separation after new slope benefit stopped. The utility mapping came from COPD evidence and the mortality relationship from an observational bronchiectasis cohort; neither has been prospectively validated for translating a brensocatib-induced FEV_1_ difference into long-term outcomes. Because the FEV_1_ and exacerbation pathways were sampled independently, their biological and statistical correlation and the risk of double counting downstream health effects remain uncertain. Priority evidence includes multi-year treatment-specific FEV_1_ data, estimates of catch-up or washout after discontinuation, and bronchiectasis-specific joint models linking FEV_1_, exacerbations, EQ-5D, and mortality. The FEV_1_-disabled analysis is a deliberately stringent structural test, not a claim that the true long-term effect is zero.

Uncertainty also surrounds treatment persistence and event utilities. Trial non-completion served as a proxy for permanent attrition, although routine-care patterns of long-term discontinuation, adherence, retreatment, and the fate of previously preserved lung function remain unknown. Acute-event QALY losses were reconstructed rather than measured prospectively in Chinese patients. Future studies should follow treatment exposure and discontinuation over time and collect EQ-5D-5L before, during, and after exacerbations so that event duration and recovery can be estimated directly.

Mortality, microbiology, cost, and safety inputs were necessarily simplified. A single NCFB standardised mortality ratio was applied across living states, and the yearbook-based age/sex mortality reconstruction used interpolation and Gompertz extrapolation rather than external calibration to Chinese bronchiectasis survival. The 20.8% PA and 5.6% NTM values were denominator-specific culture-positive proportions among tested BE-China participants, not directly observed mutually exclusive whole-cohort prevalences. The model nonetheless used them as fixed, mutually exclusive baseline weights and did not represent treatment initiation, clearance, recurrence, or co-infection. Long-term PA/NTM costs relied partly on non-Chinese sources, and the reference case assumed no net adverse-event burden. Future updates would benefit from Chinese adjusted survival models, longitudinal microbiology and treatment data, audited microcosting, and evidence on adverse-event-related resource use and discontinuation.

The healthcare-system perspective excluded productivity, caregiver time, and other non-medical consequences. The evidence search was parameter-oriented rather than a formal systematic review. Although two reviewers independently screened the deduplicated PubMed/MEDLINE and Scopus records, the search did not cover Web of Science, Embase, Dimensions, or dedicated Chinese-language databases, so small or unindexed studies may have been missed. The PubMed/MEDLINE and Scopus exports, the eight studies retained for parameter extraction, and every full-text exclusion with its reason were archived.

## 5 Conclusion

Using Chinese evidence wherever credible sources were available and testing the assumptions that still had to be transferred, the FEV_1_-inclusive base case estimated a value-based annual price of CNY 11,692 at WTP CNY 200,000/QALY (PSA median CNY 11,611). Removing all FEV_1_-linked outcome mappings lowered the estimate to CNY 990/year. Both values were far below the illustrative annual price of CNY 120,000. The central pricing implication is unchanged, although the exact estimate remains tied mainly to how long the 52-week FEV_1_ difference persists and how it affects downstream outcomes.

## Data Availability

The original contributions presented in the study are included in the article/Supplementary Material, further inquiries can be directed to the corresponding authors.
