# Supplementary material for: Value-based pricing of brensocatib for frequently exacerbating non-cystic fibrosis bronchiectasis in China: a China-adapted, FEV1-inclusive time-dependent markov analysis
Source: Front Pharmacol. 2026 Sep 25;17:1920335. doi: 10.3389/fphar.2026.1920335 (PMC13649390; doi:10.3389/fphar.2026.1920335)
Supplement: Supplementary file 1 [file Supplementaryfile1.docx]

# Supplementary Material

## S1 Decision problem and model specification

### S1.1 Base-case model specification

From the perspective of the Chinese healthcare system, the model followed the cohort over a lifetime in monthly cycles. Table S1 summarises the decision problem and base-case specification.

**Table S1 Decision problem and base-case model specification**

| **Item** | **Specification** |
| --- | --- |
| Target population | Adults with non-cystic fibrosis bronchiectasis (NCFB) and ≥2 exacerbations in the preceding 12 months; model starting age: 62 years; 56.7% female (overall BE-China cohort: 63.1 years; 58.2% female) |
| Intervention/comparator | Brensocatib 25 mg once daily plus standard care (SC) versus SC alone |
| Model structure | Seven clinical states; treatment retention, attrition, and washout tracked internally; monthly cycles; simulation to age 100 |
| Base case | The trial-observed exacerbation effect is applied, and the FEV_1_ preservation observed in the ASPEN 25-mg arm (annual decline: 62 mL under SC versus 24 mL with brensocatib) is translated into utility and mortality through external relationships |
| Key structural sensitivity | The FEV_1_-utility and FEV_1_-mortality mappings are disabled, leaving only the exacerbation benefit observed directly in the trial |
| Perspective and costs | Perspective of the Chinese healthcare system; direct medical costs only; 2025 CNY |
| Discount rate | Costs and health outcomes discounted at 4.5% annually |
| Primary outcomes | Value-based annual price (P*), QALYs, life-years, lifetime exacerbations, ICER, EVPI, and EVPPI |
| Base-case WTP | CNY 200,000/QALY (scenario range: CNY 50,000–300,000/QALY) |
| Baseline infection | PA and NTM culture-positivity proportions are used as mutually exclusive baseline weights; acquisition and clearance are simulated only in exploratory scenarios |
| Mortality structure | Age-specific background mortality multiplied by a single NCFB standardised mortality ratio (SMR); the base case also applies an FEV_1_-related mortality multiplier |
| Effect durability | After treatment discontinuation, billing stops and no further efficacy accrues; separate scenarios model persistent efficacy and 6- or 12-month washout |

*Source: authors’ model specification. Core parameters are listed in Table S4. Abbreviations: NCFB, non-cystic fibrosis bronchiectasis; SC, standard care; PA, Pseudomonas aeruginosa; NTM, nontuberculous mycobacteria; FEV_1_, forced expiratory volume in 1 s; RR, rate ratio; QALY, quality-adjusted life-year; ICER, incremental cost-effectiveness ratio; PSA, probabilistic sensitivity analysis; EVPI, expected value of perfect information; EVPPI, expected value of partial perfect information; SMR, standardised mortality ratio; WTP, willingness-to-pay threshold; CNY, Chinese yuan.*

### S1.2 Literature search and study selection

A parameter-focused search identified evidence on dipeptidyl peptidase-1 (DPP-1) inhibitors and Chinese NCFB epidemiology, costs, utilities, microbiology, and mortality. PubMed/MEDLINE and Scopus were searched through 30 June 2026; regulatory, health technology assessment (HTA), guideline, and national statistical sources were updated through 21 July 2026. One term block covered DPP-1 inhibitors, including WILLOW, ASPEN, and SAVE-BE [1-3]; a second covered Chinese NCFB parameters, including BE-China, disease-burden studies, and hospital costs [4-7]. Earlier economic evaluations [8,9], pivotal trials, regulatory and HTA documents, guidelines, reference lists, and statistical yearbooks were searched manually. Tables S2 and S3 report the full strategies.

Database searches returned 1,056 records (PubMed/MEDLINE, 370; Scopus, 686), with 752 remaining after deduplication. Eligible sources included adult NCFB trials, cohorts or registries, systematic reviews, and cost or burden studies reporting quantitative data on exacerbations, hospitalisation, PA/NTM, lung function, survival, utilities, or direct medical costs; the Chinese-parameter search was not restricted by intervention. Chinese and English reports were eligible. Studies limited to paediatric or cystic-fibrosis populations, case reports, animal studies, and sources without extractable quantitative inputs were excluded. Two reviewers independently screened titles/abstracts and full texts, resolving disagreements through discussion. After 439 title/abstract exclusions, 313 reports underwent full-text review; 8 were retained after 305 exclusions (Figure S1).

**Table S2 PubMed search strategy**

| **#** | **Query (PubMed syntax)** |
| --- | --- |
| #1 | Bronchiectasis[Mesh] |
| #2 | bronchiectasis[tiab] OR “bronchiectases”[tiab] OR “bronchiectasia”[tiab] OR “non-cystic fibrosis bronchiectasis”[tiab] OR “noncystic fibrosis bronchiectasis”[tiab] OR “non-CF bronchiectasis”[tiab] OR “NCFB”[tiab] |
| #3 | #1 OR #2 |
| #4 | brensocatib[tiab] OR “AZD7986”[tiab] OR “AZD 7986”[tiab] OR “AZD-7986”[tiab] OR “INS1007”[tiab] OR “INS 1007”[tiab] OR “INS-1007”[tiab] OR “HSK31858”[tiab] OR “HSK 31858”[tiab] OR “dipeptidyl peptidase-1 inhibitor*”[tiab] OR “dipeptidyl peptidase 1 inhibitor*”[tiab] OR “DPP-1 inhibitor*”[tiab] OR “DPP1 inhibitor*”[tiab] OR “cathepsin C inhibitor*”[tiab] |
| #5 | #3 AND #4 |
| #6 | China[Mesh] OR “China”[tiab] OR “Chinese”[tiab] OR “BE-China”[tiab] OR “SAVE-BE”[tiab] OR “Hong Kong”[tiab] OR “Taiwan”[tiab] |
| #7 | epidemiolog*[tiab] OR “prevalence”[tiab] OR “incidence”[tiab] OR “registry”[tiab] OR “registries”[tiab] OR “cohort”[tiab] OR “exacerbation*”[tiab] OR “hospitali*”[tiab] OR “cost*”[tiab] OR “economic*”[tiab] OR “expenditure*”[tiab] OR “burden”[tiab] OR “mortality”[tiab] OR “survival”[tiab] OR “quality of life”[tiab] OR “utilit*”[tiab] OR “Pseudomonas aeruginosa”[tiab] OR “nontuberculous mycobacteri*”[tiab] OR “non-tuberculous mycobacteri*”[tiab] OR “microbiolog*”[tiab] OR “aetiolog*”[tiab] OR “etiolog*”[tiab] |
| #8 | #3 AND #6 AND #7 |
| #9 | #5 OR #8 |
| #10 | #9 NOT (“animals”[Mesh] NOT “humans”[Mesh]) |
| #11 | #10 NOT (“editorial”[pt] OR “letter”[pt] OR “comment”[pt] OR “news”[pt] OR “case reports”[pt] OR “biography”[pt] OR “congress”[pt]) |
| #12 | #11 AND (English[lang] OR Chinese[lang]) |

*Source: PubMed/MEDLINE search strategy devised by the authors.*

**Table S3 Scopus search strategy**

| **#** | **Query (Scopus syntax)** |
| --- | --- |
| S1 | TITLE-ABS-KEY ( bronchiectasis OR bronchiectases OR bronchiectasia OR “non-cystic fibrosis bronchiectasis” OR “noncystic fibrosis bronchiectasis” OR “non-CF bronchiectasis” OR NCFB ) AND TITLE-ABS-KEY ( brensocatib OR AZD7986 OR “AZD 7986” OR INS1007 OR “INS 1007” OR HSK31858 OR “HSK 31858” OR “dipeptidyl peptidase-1 inhibitor*” OR “dipeptidyl peptidase 1 inhibitor*” OR “DPP-1 inhibitor*” OR “DPP1 inhibitor*” OR “cathepsin C inhibitor*” ) |
| S2 | TITLE-ABS-KEY ( bronchiectasis OR “non-cystic fibrosis bronchiectasis” OR “non-CF bronchiectasis” OR NCFB ) AND TITLE-ABS-KEY ( China OR Chinese OR “BE-China” OR “SAVE-BE” OR “Hong Kong” OR Taiwan ) AND TITLE-ABS-KEY ( epidemiolog* OR prevalence OR incidence OR registr* OR cohort OR exacerbation* OR hospitali* OR cost* OR economic* OR expenditure* OR burden OR mortality OR survival OR “quality of life” OR utilit* OR “Pseudomonas aeruginosa” OR “nontuberculous mycobacteri*” OR “non-tuberculous mycobacteri*” OR microbiolog* OR aetiolog* OR etiolog* ) |
| S3 | S1 OR S2 |
| S4 | S3 AND ( LIMIT-TO ( LANGUAGE “English” ) OR LIMIT-TO ( LANGUAGE , “Chinese” ) ) AND ( EXCLUDE ( DOCTYPE , “le” ) OR EXCLUDE ( DOCTYPE , “ed” ) OR EXCLUDE ( DOCTYPE , “no” ) OR EXCLUDE ( DOCTYPE , “er”) OR EXCLUDE ( DOCTYPE , “cp” ) ) |

*Source: Scopus search strategy devised by the authors.*

**Figure S1. Study-selection flow for model parameters. Pivotal trials, regulatory and HTA documents, clinical guidelines, and national statistics were reviewed manually and are not included in the database flow; 21 such sources are cited in the main text.**

### S1.3 Core input parameters

Table S4 presents each core input together with its uncertainty treatment, sensitivity range, evidence basis, and source. FEV_1_-linked utility and mortality mappings are active in the base case and disabled in the key structural sensitivity; PA/NTM acquisition and clearance are included only in exploratory analyses (S6).

**Table S4 Core model input parameters (base case, 2025 CNY)**

| **Parameter** | **Base value** | **Distribution** | **Sensitivity range** | **Evidence basis** | **Source** |
| --- | --- | --- | --- | --- | --- |
| **Cohort and demographics** | | | | | |
| Starting age (years) | 62 | Fixed | 60; 65 (scenario) | Local | [4] |
| Female proportion | 0.567 | Fixed | 0.489 (scenario) | Local | [4] |
| **Baseline clinical-state composition (mutually exclusive baseline weights)** | | | | | |
| S1 No chronic infection | 0.736 | Residuala | 0.65-0.82 | Local | [4] |
| S3 Chronic PA | 0.208 | Betaa | 0.15-0.27 | Local | [4] |
| S5 Chronic NTM | 0.056 | Betaa | 0.03-0.08 | Local | [4] |
| Acute exacerbations |  |  |  |  |  |
| Prevalence-weighted steady-state exacerbation-event flow under SC, λ (events/year) | 1.5 | Gamma | 1.29-2.01 | Local / calibrated | [4,2,3] |
| On-treatment exacerbation RR (brensocatib versus SC) | 0.81 | Log-normal | 0.69-0.94 | Trial | [2] |
| Exacerbation rate ratio, infected versus uninfected | 1.14 | Log-normal | 1.00-1.30 | External | [10] |
| Proportion of exacerbations that are severe (hospitalised) | 0.147 | Beta | 0.10-0.20 | Trial | [2] |
| Monthly probability of exacerbation resolution, ρ | 0.9 | Beta | 0.80-0.95 | External / assumption | — |
| **Chronic infection (fixed in the base case; dynamic acquisition and clearance included only in exploratory analyses; see S6)** | | | | | |
| Pathogen-acquisition RR (brensocatib versus SC) | 1 | Scenario | 0.7-1.00 | Trial / extrapolation | [2] |
| Monthly PA clearance probability | 0.053 | Beta | 0.03-0.08 | External | [11] |
| Monthly probability of successful NTM treatment | 0.049 | Beta | 0.03-0.07 | External | [12] |
| Monthly PA acquisition hazard | 0.0035 | Log-normal | 0.0025-0.0050 | External (exploratory) | — |
| Monthly NTM acquisition hazard | 0.0018 | Log-normal | 0.0009-0.0034 | External (exploratory) | — |
| **Mortality inputs (state-specific RRs used only in exploratory analyses)** | | | | | |
| NCFB standardised mortality ratio (SMR) | 2 | Log-normal | 1.8-3.5 | External / assumption | [4,13] |
| Mortality RR in an exacerbation state (versus stable) | 1.16 | Log-normal | 1.05-1.30 | External | [14] |
| Mortality RR for chronic PA (versus no infection) | 1.47 | Log-normal | 1.20-1.90 | External | [15] |
| Mortality RR for chronic NTM (versus no infection) | 1.47 | Perfectly correlated with PA | 1.20-1.90 | External / assumption | [15] |
| **Utilities (combined multiplicatively)** | | | | | |
| Stable NCFB | 0.719 | Beta | 0.65-0.80 | External | [14,16] |
| Non-hospitalised event QALY loss | 0.00667 | Gamma | 0.0030-0.0120 | External / derived | [14,16] |
| Chronic PA proportional utility | 0.699 | Beta | 0.62-0.78 | External | [17] |
| Chronic NTM proportional utility | 0.699 | Perfectly correlated with PA | 0.6-0.78 | External / assumption | [17] |
| Hospitalised event QALY loss | 0.00865 | Gamma | 0.0040-0.0180 | External / derived | [14,16,18] |
| Costs (2025 CNY) |  |  |  |  |  |
| Monthly cost, stable NCFB | 361.6 | Gamma | 241-723 | Local | [5] |
| Cost per non-hospitalised exacerbation | 542.4 | Gamma | 361-1,085 | Local | [6] |
| Cost per hospitalised severe exacerbation | 11000 | Gamma | 7,000-16,000 | Local | [6,7] |
| Monthly cost, chronic PA | 1500 | Gamma | 800-3,000 | External | [19] |
| Monthly cost, chronic NTM | 2200 | Gamma | 1,200-3,500 | External | [20] |
| **Discounting and treatment attrition** | | | | | |
| Discount rate (costs and health outcomes) | 4.5%/year | Fixed | 0%–5% (scenario) | Local | [21] |
| Annualised treatment attrition proxy | 0.133 | Beta | 0.10-0.20 | Trial / assumption | [2] |
| **Lung-function preservation (FEV_1_; included in the base case; FEV_1_-disabled analysis is the key sensitivity)** | | | | | |
| Baseline FEV_1_ (L; % predicted) | 1.81 (71.0%) | Fixed | 60%-80% | Local | [4] |
| Annual FEV_1_ decline under SC (mL/year) | 62 | Fixed | 45-62 (scenario) | Trial / external | [2] |
| Between-arm difference in annual FEV_1_ decline (SC -brensocatib; mL/year) | 38 | Normalb | 11-65 | Trial | [2] |
| Annual FEV_1_ decline after discontinuation (mL/year) | 62 | Fixed | — | Structural | procedure |
| Mortality RR per 10-point fall in FEV_1_ (% predicted) | 1.2 | Log-normal | 1.16-1.23 | External / assumption | [13] |
| FEV_1_ → utility coefficient β (per 10 mL) | 0.0004 | Log-normal | 0.0002-0.0006 | External / assumption | [22] |
| Natural-history framework (COPD simulation model) | — | — | — | External | [23] |

*Note: The Distribution column describes the treatment of uncertainty. Named distributions are sampled in the PSA whenever the corresponding structure is active; Fixed inputs remain at their base values, Derived inputs are calculated from sampled quantities, and Scenario inputs are used only in the specified structural analyses. The Evidence basis column classifies inputs as local, trial, external, or structural.*

*a Baseline composition is sampled jointly. PA and NTM shares are drawn independently from Beta distributions calibrated to the stated base values and 95% ranges. If their sum exceeds 0.95, both shares are rescaled proportionally; the infection-negative share is the residual 1 − PA − NTM. c The exacerbation-, PA-, and NTM-specific mortality RRs are used only in the exploratory normalised state-mortality analysis (S7; Table S8), not in the reference structure.*

*b The PSA samples the between-arm annual FEV_1_ decline difference from a Normal distribution centred at 38 mL/year (95% CI 11–65) and truncated to [−20, 62]. Brensocatib decline is calculated as 62 mL/year minus this draw, giving a base value of 24 mL/year. The FEV_1_–utility and FEV_1_–mortality coefficients are sampled from the stated Log-normal distributions in the base case and set to zero only in the prespecified FEV_1_-disabled sensitivity. PA/NTM utility and exploratory mortality inputs are perfectly correlated, with NTM taking the PA draw. Source: compiled from the cited literature and model specification.*

## S2 Background mortality and age handling

Age- and sex-specific all-cause mortality was reconstructed from the 2025 China Health Statistics Yearbook [24] and cross-checked against national trends reported by Ling et al. [25], which did not supply numerical model inputs. The base case began at age 62 with 56.7% women [4]; scenarios used starting ages of 60 or 65 years and the 49.0% female share reported in the 2025 national statistical communiqué [26]. Rates for ages 50-84 were weighted by cohort sex, interpolated log-linearly between age groups, and extrapolated at older ages with a Gompertz function. Annual death probabilities were converted to continuous hazards, updated monthly by attained age, and multiplied by the NCFB SMR of 2.0 (range 1.8-3.5), informed by BE-China [4] and Loebinger et al. [13]. Selected single-year probabilities are shown in Table S5.

**Table S5 Single-year background probabilities of death for the target cohort (reconstructed from the yearbook; selected ages)**

| Age | Annual probability of death, q | Per 1,000 |
| --- | --- | --- |
| 50 | 0.00358 | 3.58 |
| 55 | 0.00434 | 4.34 |
| 60 | 0.00649 | 6.49 |
| 62 | 0.0083 | 8.3 |
| 65 | 0.01136 | 11.36 |
| 70 | 0.01876 | 18.76 |
| 75 | 0.03111 | 31.11 |
| 80 | 0.05422 | 54.22 |
| 85 | 0.09854 | 98.54 |
| 90 | 0.1791 | 179.1 |
| 95 | 0.32551 | 325.51 |
| 100 | 0.59162 | 591.62 |

*Source: 2025 China Health Statistics Yearbook [24] and authors’ calculations. The values shown are general-population background probabilities; the disease SMR is applied when patients enter the NCFB model.*

## S3 Health states, transitions, and the exacerbation event flow

Exacerbations arise only from stable states S1, S3, and S5. Monthly onset probabilities were calibrated to a prevalence-weighted SC target of 1.50 events/year, an infected-to-uninfected rate ratio of 1.14, and a monthly resolution probability of 0.90. The infection-negative subgroup retains the uninfected rate implied by this weighted calibration; the “No chronic PA/NTM module” instead recalibrates its single process directly to 1.50/year. Each event, cost, and QALY decrement is recorded once at onset.The seven clinical states are defined in Table S6.

The 1.50/year target is a steady-state event flow, not the realised first-year rate. Because all patients enter a stable state, year 1 is transitional: SC produces 1.517 events/person-year. Applying RR 0.81 to retained on-treatment stratum-specific rates before nonlinear conversion yields 1.244 events/person-year and a whole-arm ratio of 0.820 after attrition; with attrition disabled, the ratio is 1.226/1.517 = 0.808. The modest departure from 0.81 reflects the initial-state transient and rate-to-probability conversion. No half-cycle correction was applied.

**Table S6 Definitions and attributes of the seven clinical states**

| **State** | **Definition** | **Attributes** |
| --- | --- | --- |
| S1 | Stable NCFB, no chronic infection | Base utility and stable-state cost |
| S2 | No chronic infection + exacerbation | Event-carrying state; background utility as in S1; acute loss counted once at onset |
| S3 | Chronic PA, no exacerbation | Base utility × PA proportional utility; monthly PA cost added |
| S4 | Chronic PA + exacerbation | Event-carrying state; background utility as in S3; acute loss counted once at onset |
| S5 | Chronic NTM, no exacerbation | Base utility × NTM proportional utility; monthly NTM cost added |
| S6 | Chronic NTM + exacerbation | Event-carrying state; background utility as in S5; acute loss counted once at onset |
| S7 | Death | Absorbing state |

*Source: authors’ model structure.*

## S4 Utility reconstruction of exacerbations

The 0.758 proportional utility reported for frequent exacerbators [14] was converted to a one-off 14-day loss rather than applied across an entire monthly exacerbation state. Hospitalised events also use the 0.905 multiplier [18].

Chronic PA and NTM reduce background-state utility, whereas exacerbations impose the same absolute event loss in every infection stratum. The reconstructed losses are 0.00667 QALY for non-hospitalised events and 0.00865 QALY for hospitalised events (severity-weighted mean, 0.00696; Table S7). In the PSA, these event losses are sampled from Gamma distributions. They are not derived from the 0.758 and 0.905 reconstruction anchors.

**Table S7 QALY loss per one-off exacerbation event**

| **Parameter** | **Base value** | **Calculation / use** |
| --- | --- | --- |
| Stable NCFB utility | 0.719 | Starkie mapping anchor |
| Acute-phase proportional utility | 0.758 | Reconstruction anchor only; not applied to current-state utility |
| Hospitalisation multiplier | 0.905 | Reconstruction anchor for hospitalised events |
| Non-hospitalised event loss | 0.00667 QALY | (0.719 − 0.719×0.758) × 14/365.25 |
| Hospitalised event loss | 0.00865 QALY | (0.719 − 0.719×0.758×0.905) × 14/365.25 |
| Severity-weighted event loss | 0.00696 QALY | Hospitalisation share: 14.7%; the same absolute loss is applied to events in all infection strata |

*Source: the cited utility studies and authors’ calculations.*

## S5 Base case, treatment attrition, effect durability, and lung-function preservation

Treatment retention, attrition, and washout are identical in the base case and FEV_1_-disabled sensitivity; only the FEV_1_ outcome mappings differ. Drug costs accrue while patients remain on treatment, and the 13.3% non-completion at 52 weeks in ASPEN is used as the annual attrition proxy [2].

While patients remain on treatment, brensocatib applies an exacerbation RR of 0.81 and annual FEV1 decline of 24 mL, versus 62 mL under SC. After attrition, decline returns to the SC rate, but accumulated FEV1 separation is retained. FEV1 is updated monthly with a 0.50-L floor. Each 10 mL preserved adds 0.0004 to utility [22], while each 10-percentage-point decline in FEV1% predicted multiplies mortality by 1.20 [13]. These external mappings are embedded in the natural-history framework adapted from Borg et al. [23], not directly observed as long-term ASPEN outcomes.

The model tracks one arm-level cohort-mean FEV_1_ trajectory, F(t). At entry, F(0) = 1.81 L corresponds to 71.0% predicted, implying a reference-normal volume of 2.55 L. Thus FEV_1_% predicted(t) = 71.0 + 100 × [F(t) - 1.81]/2.55, and the mortality multiplier is 1.20^[(71.0 − FEV_1_% predicted(t))/10]. The multiplier equals 1.0 at entry and is applied to the age- and sex-specific background hazard after the common NCFB SMR of 2.0; it therefore captures within-model decline from baseline, not deviation from 100% predicted.

The monthly utility adjustment, 0.0004 × [F(t) - 1.81]/0.01, is added after applying the PA/NTM utility multiplier. FEV_1_ is bounded below at 0.50 L, and final state utility is constrained to [0,1]. No upper FEV_1_ bound is required because all modelled trajectories are non-improving.

In the retained-only base case, billing and future exacerbation-rate and FEV_1_-slope benefits cease at attrition, while accumulated FEV_1_ separation remains. Six- and 12-month washout scenarios attenuate both future effects linearly but retain that separation. Under persistent post-attrition efficacy, billing stops but both effects continue among surviving former users. A separate calendar-time waning scenario attenuates only the exacerbation RR. The PSA samples the between-arm FEV_1_ slope difference and both mappings; the key structural sensitivity disables the mappings while retaining the trial-observed exacerbation effect.

## S6 Chronic infection natural history

BE-China reported PA and NTM positivity of 20.8% and 5.6%, respectively, using different culture denominators [4]. The reference model treats these as mutually exclusive baseline weights, keeps infection status fixed, and excludes co-infection. Acquisition and clearance are activated only in exploratory analyses (Table S8).

## S7 Mortality structure and control of double-counting

The reference structure applies a uniform SMR of 2.0 to all living states and does not additionally apply long-term mortality RRs for exacerbation, PA, or NTM. This approach avoids double-counting overall bronchiectasis risk and prevents long-term prognostic associations from being attributed to a single acute month. An exploratory scenario applies normalised state-specific RRs while preserving the same cohort-average mortality multiple (Table S8).

## S8 Costs and adverse events

Costs are detailed in Table S4. Exacerbations incur a one-off cost at onset (severity-weighted mean, CNY 2,079.7/event); long-term PA and NTM management costs come from external studies and are examined as structural uncertainty. Because overall safety was similar between ASPEN arms, the base case assigns no net adverse-event cost or QALY loss; Table S8 presents a conservative alternative.

## S9 Base-case results, threshold pricing, and uncertainty

Tables 1 and 2 in the main text report base-case costs and outcomes. Discounted on-treatment exposure for threshold pricing was D = 4.5825 person-years, and P* = (WTP × ΔQ - ΔCother)/D. At WTP values of CNY 50,000, 100,000, 200,000, and 300,000/QALY, P* was CNY 2,655, 5,667, 11,692, and 17,717/year, respectively; the FEV_1_-disabled value at the primary threshold was CNY 990/year. P* is a modelled ceiling, not a reimbursement offer.

In paired PSA, median P* was CNY 11,611/year (95% simulation interval 4,145-19,324) in the base case and CNY 954/year (267-1,849) with FEV_1_ mappings disabled. At CNY 120,000/year, none of 10,000 draws was cost-effective at WTP up to CNY 300,000/QALY; the reported 0% is the empirical probability at the finite simulation resolution.

## S10 Main scenario analysis results

Table S8 presents all deterministic structural and scenario analyses. Local demographic inputs and the baseline exacerbation rate changed P* little; FEV_1_-linked outcomes and post-attrition efficacy were the principal drivers, and every estimate remained below CNY 120,000/year. Baseline infection status produced a moderate range (CNY 7,780 for NTM-positive to CNY 12,918 for infection-negative), reflecting differences in background utility, chronic-management costs, and exacerbation burden rather than treatment-effect modification. Because the reference model applies a uniform SMR, ΔLY was 0.3432 in all three infection-subgroup reruns. With exacerbation efficacy removed, the FEV_1_-only pathway yielded P* = CNY 10,702 and +1.0097 lifetime exacerbations, as longer survival increased time at risk.

**Table S8 All deterministic structural and scenario analyses (FEV_1_-inclusive base case)**

| **Scenario** | **ΔQALY** | **ΔLY** | **Δ exacerbations** | **P* (CNY/year)** | **ICER at CNY 120,000 (CNY/QALY)** |
| --- | --- | --- | --- | --- | --- |
| FEV_1_-inclusive base case | 0.2761 | 0.3432 | -0.5749 | 11,692 | 1,997,665 |
| FEV_1_ outcome mappings disabled | 0.0093 | 0 | -1.636 | 990 | 60,142,140 |
| Efficacy persists after attrition | 0.5807 | 0.7312 | -1.8192 | 24,432 | 960,496 |
| 6-month linear washout | 0.286 | 0.3554 | -0.595 | 12,106 | 1,929,592 |
| 12-month linear washout | 0.2956 | 0.3673 | -0.615 | 12,507 | 1,867,852 |
| Annual attrition: 10% | 0.3222 | 0.4018 | -0.7253 | 11,572 | 2,018,783 |
| Annual attrition: 20% | 0.2097 | 0.2593 | -0.3855 | 11,876 | 1,966,250 |
| Exacerbation RR declining to 1 by year 5 | 0.272 | 0.3432 | 0.2444 | 11,243 | 2,032,486 |
| Lower exacerbation rate, 1.29/year | 0.2753 | 0.3432 | -0.494 | 11,608 | 2,004,116 |
| Higher exacerbation rate, 2.01/year | 0.278 | 0.3432 | -0.7721 | 11,897 | 1,982,107 |
| Optimistic exacerbation RR, 0.69 | 0.2818 | 0.3432 | -1.5749 | 12,317 | 1,950,898 |
| Conservative exacerbation RR, 0.94 | 0.2699 | 0.3432 | 0.5091 | 11,015 | 2,050,613 |
| Exploratory dynamic infection: pathogen RR = 1 | 0.2887 | 0.3432 | -0.5694 | 12,481 | 1,906,760 |
| Exploratory dynamic infection: pathogen RR = 0.78 | 0.3046 | 0.3432 | -0.5855 | 13,516 | 1,802,006 |
| Normalised state-mortality RRs | 0.2785 | 0.3462 | -0.5667 | 11,814 | 1,978,061 |
| Starting age 60 | 0.2855 | 0.3512 | -0.5606 | 11,802 | 1,979,841 |
| Starting age 65 | 0.257 | 0.3236 | -0.605 | 11,366 | 2,055,648 |
| General-population female share, 49.0% | 0.275 | 0.3429 | -0.5703 | 11,712 | 1,993,579 |
| Conservative adverse-event burden | 0.2752 | 0.3432 | -0.5749 | 11,552 | 2,005,984 |
| Baseline infection-negative | 0.2955 | 0.3432 | -0.5543 | 12,918 | 1,860,569 |
| Baseline PA-positive | 0.222 | 0.3432 | -0.6324 | 8,409 | 2,503,728 |
| Baseline NTM-positive | 0.222 | 0.3432 | -0.6324 | 7,780 | 2,516,716 |
| No chronic PA/NTM module | 0.2957 | 0.3432 | -0.5749 | 12,939 | 1,859,130 |
| FEV_1_ pathway with no exacerbation efficacy | 0.267 | 0.3432 | 1.0097 | 10,702 | 2,075,897 |

*Source: model calculations. Negative incremental exacerbations indicate events averted; positive values indicate additional events arising from longer survival. Infection-subgroup reruns assign the entire cohort to the specified baseline stratum while retaining uniform-SMR mortality. The infection-negative rerun retains the uninfected rate implied by the weighted 1.50/year calibration (1.4465/year), whereas the “No chronic PA/NTM module” removes the infection states and recalibrates a single process directly to 1.50/year; their P* values are therefore close but not identical (CNY 12,918 versus 12,939). The “FEV_1_ pathway with no exacerbation efficacy” sets RR = 1.00 and retains only the FEV_1_ mappings.*

### S10.1 Lung-function pathway decomposition and structural checks

Disabling both FEV_1_ mappings made the base-case model identical to the standalone FEV_1_-disabled structure (P* = CNY 990). Utility-only and mortality-only mappings produced P* values of CNY 3,681 and CNY 9,797; the complete pathway yielded CNY 11,692. Varying the ASPEN FEV_1_ slope difference across its 95% CI gave CNY 4,046–18,615. Because FEV_1_-related survival prolongs time at risk, pathway contributions are not additive, and lifetime exacerbations should be interpreted alongside life-years. The pathway decomposition and nested structural checks are summarised in Figure S2.

With both mappings disabled, the trial-based exacerbation RR drove the one-way analysis: P* ranged from CNY 313 to 1,615/year, approximately 2.7 times the range of the next-ranked parameter (Table S9).

**Table S9 Deterministic sensitivity analysis of P* with FEV_1_-linked outcome mappings disabled**

| **Parameter** | **Low input** | **P* at low** | **High input** | **P* at high** | **Absolute range** |
| --- | --- | --- | --- | --- | --- |
| Trial-based exacerbation RR | 0.69 | 1,615 | 0.94 | 313 | 1,302 |
| SC annual exacerbation target | 1.29 | 851 | 2.01 | 1,328 | 477 |
| Non-hospitalised event QALY loss | 0.003 | 812 | 0.012 | 1,250 | 438 |
| Hospitalised event cost (CNY) | 7,000 | 823 | 16,000 | 1,200 | 377 |
| Hospitalised exacerbation fraction | 0.1 | 845 | 0.2 | 1,154 | 310 |
| Non-hospitalised event cost (CNY) | 361 | 946 | 1,085 | 1,122 | 176 |
| Hospitalised event QALY loss | 0.004 | 951 | 0.018 | 1,069 | 117 |
| Baseline PA prevalence | 0.15 | 982 | 0.27 | 999 | 16 |
| Baseline NTM prevalence | 0.03 | 987 | 0.08 | 993 | 7 |
| Annual treatment attrition | 0.1 | 990 | 0.2 | 991 | 2 |

*Source: deterministic model calculations at WTP CNY 200,000/QALY. Base P* in the FEV_1_-disabled analysis = CNY 990/year. Values are rounded to the nearest CNY.*

**Figure S2. Lung-function pathway decomposition and structural checks. Panel A presents incremental QALYs; Panel B presents P* at WTP CNY 200,000/QALY. Disabling both FEV_1_ mappings exactly reproduces the prespecified FEV_1_-disabled structure. Source: model calculations.**

## S11 Model verification and computational checks

Independent implementations reproduced both the base case and the FEV_1_-disabled structure. Transition conservation, event accounting, FEV_1_ trajectories, treatment retention, and the nested mapping-off check were verified (Table S10). These are internal numerical checks rather than external calibration [27]. EVPPI was estimated using a regression-based metamodel [28].

**Table S10 Verification and computational check results for the base case and the FEV_1_-disabled structure**

| **Check** | **Result** | **Interpretation** |
| --- | --- | --- |
| Maximum row-sum error of the transition matrix | 0 | Target: 0 |
| One-off exacerbation-event cost | CNY 2,079.7 | Charged once at event onset |
| One-off event QALY loss | 0.00696 | Deducted once at event onset |
| Year-1 exacerbation rate, SC / brensocatib | 1.517 / 1.244 | Transient realised rates; 1.50/year is the SC steady-state target, and the whole-arm year-1 rate ratio is 0.820 after attrition |
| On-treatment share among survivors at month 12 | 0.867 | Annualised attrition proxy: 13.3% |
| Base-case median survival, SC / brensocatib | 13.38 / 14.16 years | FEV_1_ pathway enabled |
| Month-12 FEV_1_, SC / brensocatib | 1.748 / 1.784 L | Base case |
| Month-60 FEV_1_, SC / brensocatib | 1.500 / 1.637 L | Base case |
| Month-60 PA / NTM (survivors) | 20.8% / 5.6% | Fixed-infection structure |
| Maximum numerical discrepancy between independent programs | 2.15e-09 / 1.63e-09 | Base case / FEV_1_-disabled structure |
| Nested check after disabling the mappings | 0 | Exactly reproduces the FEV_1_-disabled structure |
| Alive at age 100, SC / brensocatib | Base: 1.67e−13 / 2.92e−13; FEV_1_-disabled: 7.17e−6 / 7.17e−6 | All values <0.001%; the post-age-100 tail is negligible |

*Source: model verification and computational checks.*

## S12 Value-of-information analysis

At CNY 120,000/year, every finite-sample PSA draw selected SC, yielding an empirical EVPI of CNY 0. Near the probabilistic decision price (about CNY 11,691/year), EVPI rose to CNY 7,432/person. Grouped EVPPI (main-text Figure 7B) was highest for the FEV_1_ treatment effect (CNY 5,313), followed by event disutility and other utilities (CNY 968), FEV_1_ outcome mappings (CNY 953), exacerbation efficacy and rates (CNY 533), direct medical costs (CNY 295), baseline infection composition (CNY 235), attrition (CNY 139), and mortality (CNY 98); groups are not additive. The non-zero EVPPI values for infection composition and the three FEV_1_ parameters reflect their inclusion in the base-case PSA.

**References**

[1] Chalmers, JD, Haworth, CS, Metersky, ML, et al. Phase 2 Trial of the DPP-1 Inhibitor Brensocatib in Bronchiectasis. NEW ENGL J MED. 2020; 383 (22): 2127-2137. doi: 10.1056/NEJMoa2021713
[2] Chalmers, JD, Burgel, PR, Daley, CL, et al. Phase 3 Trial of the DPP-1 Inhibitor Brensocatib in Bronchiectasis. N Engl J Med. 2025; 392 (16): 1569-1581. doi: 10.1056/NEJMoa2411664
[3] Zhong, NS, Qiu, R, Cao, J, et al. Effects of the DPP-1 inhibitor HSK31858 in adults with bronchiectasis in China (SAVE-BE): a phase 2, multicentre, double-blind, randomised, placebo-controlled trial. Lancet Respir Med. 2025; 13 (5): 414-424. doi: 10.1016/S2213-2600(25)00019-0
[4] Xu, JF, Zheng, HZ, Lu, HW, et al. Baseline characteristics of patients in the Chinese Bronchiectasis Registry (BE-China): a multicentre prospective cohort study. LANCET RESP MED. 2025; 13 (2): 166-176. doi: 10.1016/S2213-2600(24)00364-3
[5] Feng, J, Sun, L, Sun, X, et al. Increasing prevalence and burden of bronchiectasis in urban Chinese adults, 2013-2017: a nationwide population-based cohort study. Respir Res. 2022; 23 (1): 111. doi: 10.1186/s12931-022-02023-8
[6] Ma, Y, Liu, D, Ji, Y, et al. Clinical features related to hospital expenses for non-cystic fibrosis bronchiectasis in China. J INT MED RES. 2020; 48 (6): 300060520931616. doi: 10.1177/0300060520932116
[7] Lei, C, Zeng, Z, Chen, F, et al. Eosinophilic bronchiectasis increases length and cost of hospitalization: a retrospective analysis in a hospital of southern China from 2012 to 2020. BMC Pulm Med. 2024; 24 (1): 98. doi: 10.1186/s12890-024-02912-2
[8] Wasfy JH, Kim K, Touchette DR, McKenna A, Richardson M, HerceHagiwara B, Kim S, Philips M, Ollendorf D. Brensocatib for Non-Cystic Fibrosis Bronchiectasis: Effectiveness and Value; Final Report. Institute for Clinical and Economic Review, October 30, 2025.https://icer.org/wp-content/uploads/2025/10/ICER_NCFB_Final-Report_ForPublication_103025.pdf.
[9] McKenna, A, Wasfy, JH, Kim, K, et al. The effectiveness and value of brensocatib for the treatment of non-cystic fibrosis bronchiectasis. J MANAG CARE SPEC PH. 2026; 32 (3): 392-397. doi: 10.18553/jmcp.2026.32.3.392
[10] Araújo, D, Shteinberg, M, Aliberti, S, et al. The independent contribution of Pseudomonas aeruginosa infection to long-term clinical outcomes in bronchiectasis. EUR RESPIR J. 2018; 51 (2): doi: 10.1183/13993003.01953-2017
[11] Conceição, M, Shteinberg, M, Goeminne, P, et al. Eradication treatment for Pseudomonas aeruginosa infection in adults with bronchiectasis: a systematic review and meta-analysis. EUR RESPIR REV. 2024; 33 (171): doi: 10.1183/16000617.0178-2023
[12] Kwak, N, Dalcolmo, MP, Daley, CL, et al. M ycobacterium abscessus pulmonary disease: individual patient data meta-analysis. EUR RESPIR J. 2019; 54 (1): doi: 10.1183/13993003.01991-2018
[13] Loebinger, MR, Wells, AU, Hansell, DM, et al. Mortality in bronchiectasis: a long-term study assessing the factors influencing survival. EUR RESPIR J. 2009; 34 (4): 843-9. doi: 10.1183/09031936.00003709
[14] Chalmers, JD, Aliberti, S, Filonenko, A, et al. Characterization of the "Frequent Exacerbator Phenotype" in Bronchiectasis. AM J RESP CRIT CARE. 2018; 197 (11): 1410-1420. doi: 10.1164/rccm.201711-2202OC
[15] Jacobs, DM, Ochs-Balcom, HM, Noyes, K, et al. Impact of Pseudomonas aeruginosa Isolation on Mortality and Outcomes in an Outpatient Chronic Obstructive Pulmonary Disease Cohort. OPEN FORUM INFECT DI. 2020; 7 (1): ofz546. doi: 10.1093/ofid/ofz546
[16] Starkie, HJ, Briggs, AH, Chambers, MG, et al. Predicting EQ-5D values using the SGRQ. VALUE HEALTH. 2011; 14 (2): 354-60. doi: 10.1016/j.jval.2010.09.011
[17] Chalmers, JD, Goeminne, P, Aliberti, S, et al. The bronchiectasis severity index. An international derivation and validation study. AM J RESP CRIT CARE. 2014; 189 (5): 576-85. doi: 10.1164/rccm.201309-1575OC
[18] Camac, ER, Voelker, H, Criner, GJ. Impact of COPD exacerbations leading to hospitalization on general and disease-specific quality of life. RESP MED. 2021; 186 106526. doi: 10.1016/j.rmed.2021.106526
[19] Blanchette, CM, Noone, JM, Stone, G, et al. Healthcare Cost and Utilization before and after Diagnosis of Pseudomonas aeruginosa among Patients with Non-Cystic Fibrosis Bronchiectasis in the U.S. Med Sci (Basel). 2017; 5 (4): doi: 10.3390/medsci5040020
[20] Marras, TK, Mirsaeidi, M, Chou, E, et al. Health Care Utilization and Expenditures Following Diagnosis of Nontuberculous Mycobacterial Lung Disease in the United States. J MANAG CARE SPEC PH. 2018; 24 (10): 964-974. doi: 10.18553/jmcp.2018.18122
[21] Wu J, Liu GE. China Guidelines for Pharmacoeconomic Evaluations 2025 Chinese-English Version. China Planning Press; 2025.
[22] Rutten-van Mölken, MP, Oostenbrink, JB, Tashkin, DP, et al. Does quality of life of COPD patients as measured by the generic EuroQol five-dimension questionnaire differentiate between COPD severity stages? CHEST. 2006; 130 (4): 1117-28. doi: 10.1378/chest.130.4.1117
[23] Borg, S, Ericsson, A, Wedzicha, J, et al. A computer simulation model of the natural history and economic impact of chronic obstructive pulmonary disease. VALUE HEALTH. 2004; 7 (2): 153-67. doi: 10.1111/j.1524-4733.2004.72318.x
[24] National Health Commission of the People’s Republic of China. China Health Statistics Yearbook 2025. Peking Union Medical College Press; 2025. Chinese.
[25] Ling X, Lin XW, Zhang Y, et al. Analysis on incidence of death in urban and rural residents in China, 2015–2021. Disease Surveillance. 2024;39(3):369-375. doi:10.3784/jbjc.202305090206. Chinese.
[26] National Bureau of Statistics of China. Statistical Communiqué of the People’s Republic of China on the 2025 National Economic and Social Development. National Bureau of Statistics of China, February 28, 2026. https://www.stats.gov.cn/english/PressRelease/202602/t20260228_1962661.html
[27] Eddy, DM, Hollingworth, W, Caro, JJ, et al. Model transparency and validation: a report of the ISPOR-SMDM Modeling Good Research Practices Task Force--7. VALUE HEALTH. 2012; 15 (6): 843-50. doi: 10.1016/j.jval.2012.04.012
[28] Strong, M, Oakley, JE, Brennan, A. Estimating multiparameter partial expected value of perfect information from a probabilistic sensitivity analysis sample: a nonparametric regression approach. MED DECIS MAKING. 2014; 34 (3): 311-26. doi: 10.1177/0272989X13505910
